# Supplementary material for: Genetic Diversity of Plasmodium falciparum in Korhogo Health District, Northern Côte d’Ivoire
Source: Trop Med Infect Dis. 2025 Sep 7;10(9):255. doi: 10.3390/tropicalmed10090255 (PMC12474276; doi:10.3390/tropicalmed10090255)
Supplement: Supplementary file 1 [file tropicalmed-10-00255-s001.zip › tropicalmed-3826378-supplementary.pdf]

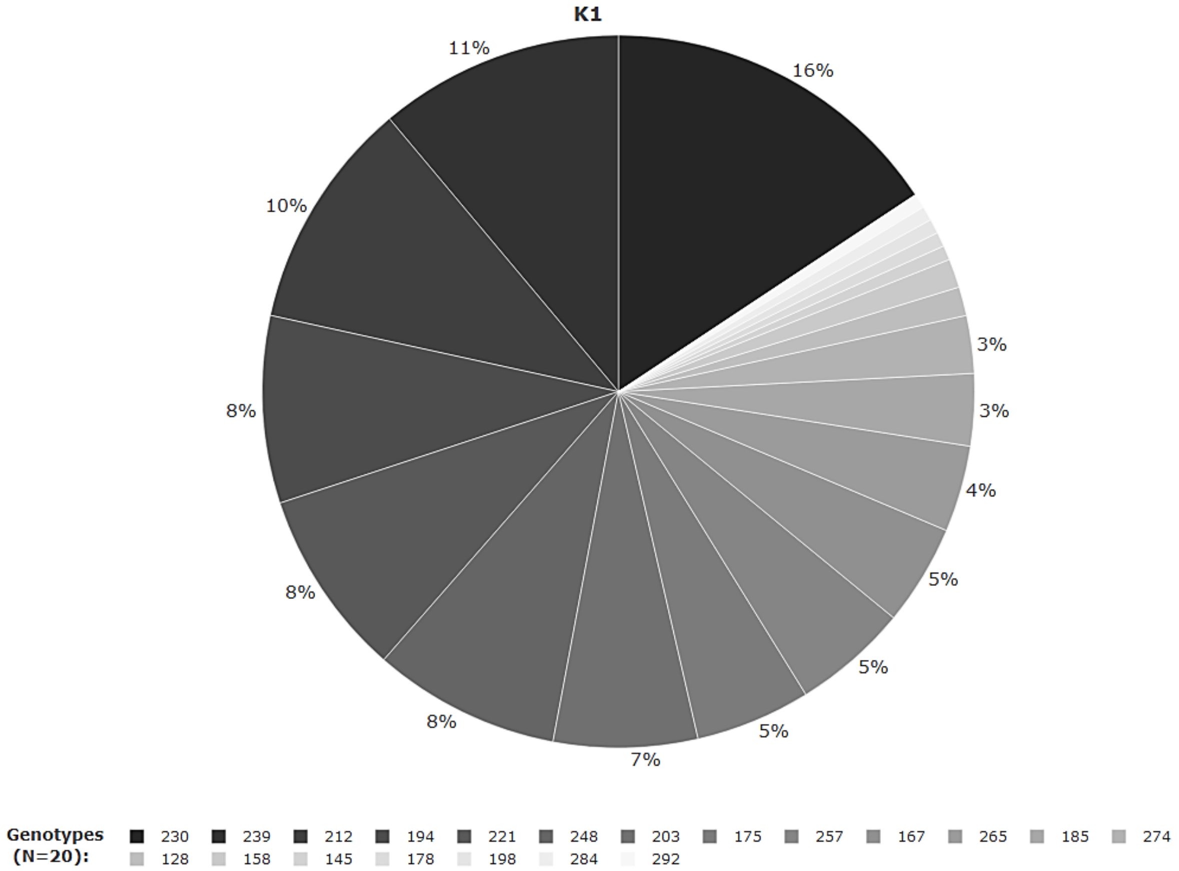

Figure S1. K1 fragment sizes and frequencies. Twenty (20) genotypes are identified based on the fragment sizes. From left to right, the genotypes are listed in order of decreasing frequency. A genotype 230 bp is the most abundant with a frequency of 16%, followed by the genotypes 239 and 212 bp with frequencies of 11 and 10% respectively. The frequencies are found at 8% for 03 genotypes (194, 221, and 248 bp), 7% for 01 genotype (203 bp), 5% for 03 genotypes (175, 257 and 167 bp), 4% for 01 genotype (265 bp) and 3% for 02 genotypes (185 and 274 bp). The others occur at a frequency < 3%.

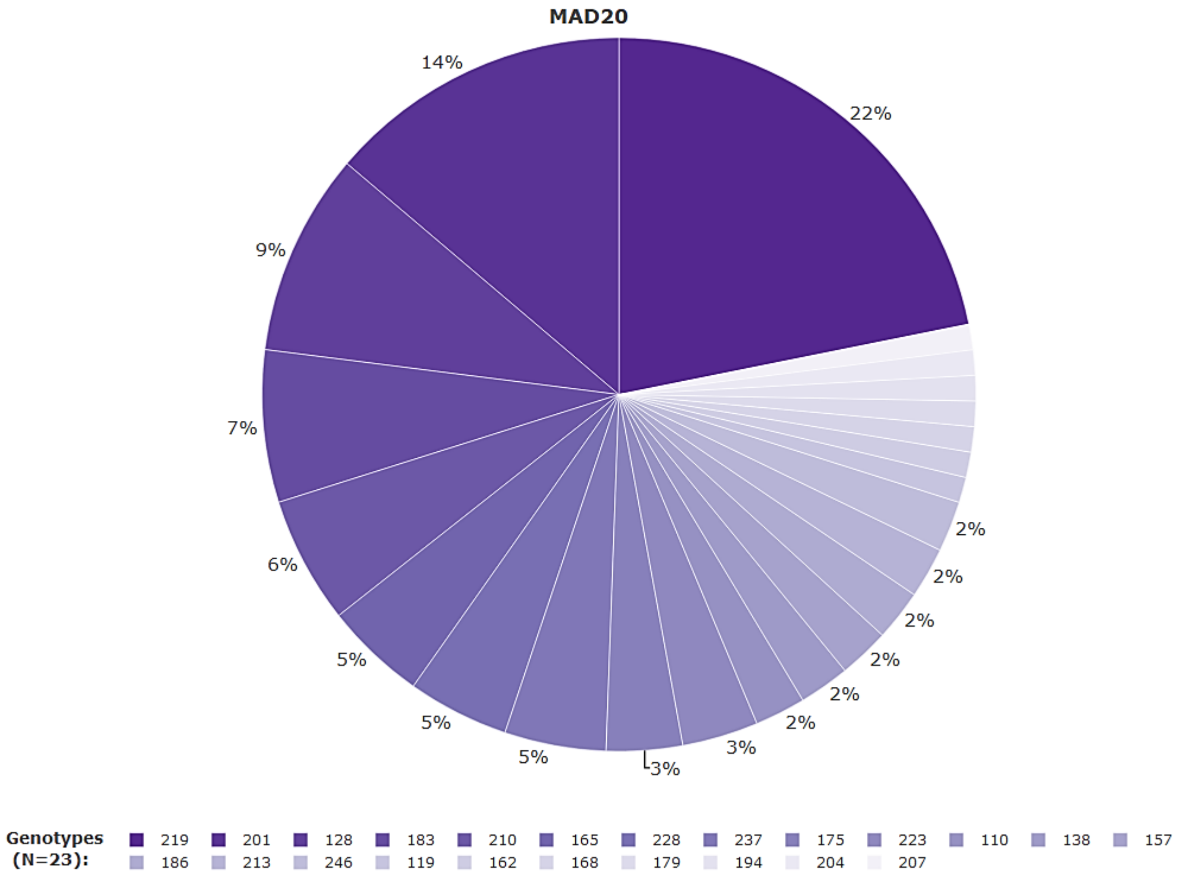

Figure S2. MAD20 fragment sizes and frequencies. Twenty-three (23) genotypes are identified based on the fragment sizes. From left to right, the genotypes are listed in order of decreasing frequency. A genotype 219 bp is the most abundant with a frequency of 22%, followed by a genotype 201 bp with a frequency of 14 %. The genotypes 128, 183 and 210 bp occur at the frequencies of 9, 7, and 6% respectively. The frequencies are found at 5% for 03 genotypes (165, 228 and 237 bp), 3% for 02 genotypes (175 and 223 bp) and 2% for 06 genotypes (110, 138, 157, 186, 213 and 246 bp). The other genotypes occur at a frequency < 2%.

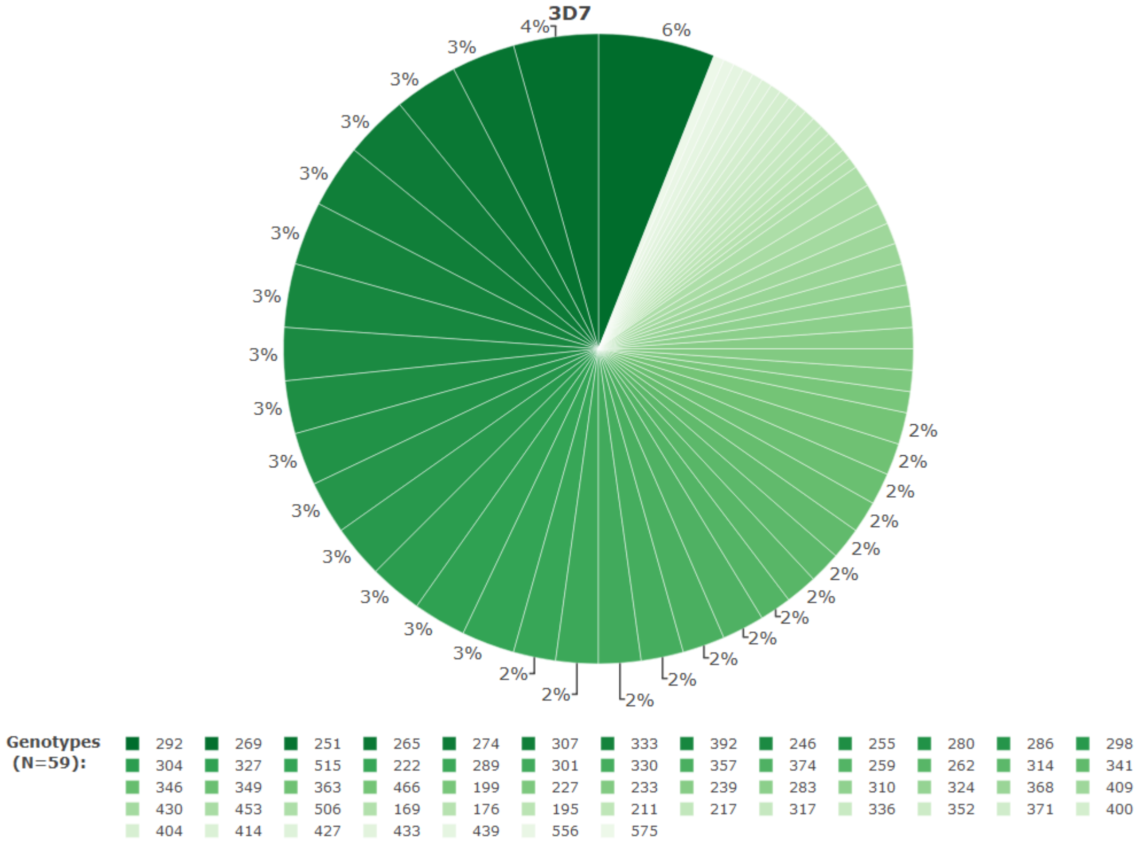

Figure S3. 3D7 fragment sizes and frequencies. Fifty-nine (59) genotypes are identified based on the fragment sizes. From left to right, the genotypes are listed in order of decreasing frequency. A genotype 292 bp is the most abundant with a frequency of 6%. The frequencies are found at 4% for 01 genotype (269 bp), 3% for 14 genotypes (251, 265, 274, 307, 333, 392, 246, 255, 280, 286, 298, 304, 327, 515 bp) and 2% for 14 genotypes (222, 289, 301, 330, 357, 374, 259, 262, 314, 341, 346, 349, 363 and 466 bp). The others occur at a frequency < 2%.

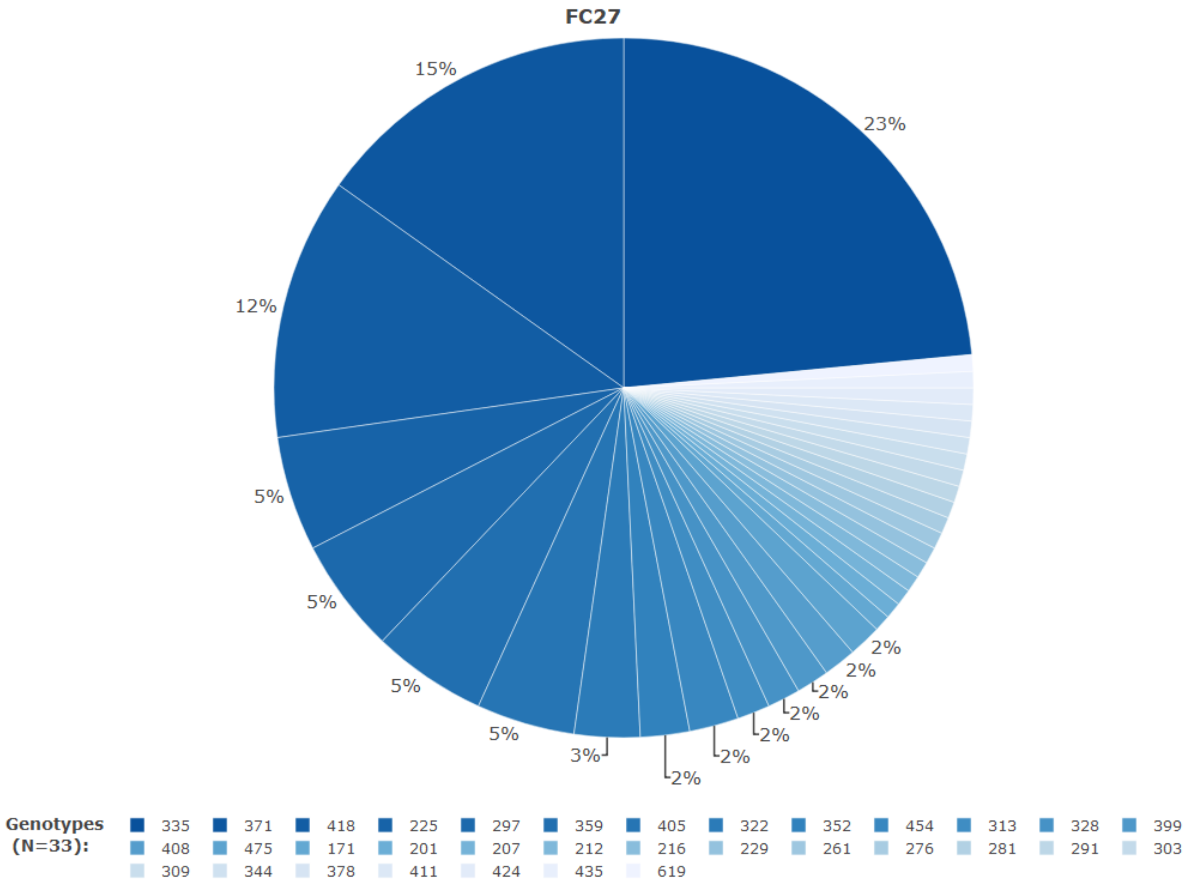

Figure S4. FC27 fragment sizes and frequencies. Thirty-three (33) genotypes are identified based on the fragment sizes. From left to right, the genotypes are listed in order of decreasing frequency. A genotype 335 bp is the most abundant with a frequency of 23%, followed by the genotypes 371 and 418 bp with frequencies of 15 and 12% respectively. The frequencies are found at 5% for 04 genotypes (225, 297, 359, and 405 bp), 3% for 01 genotype (322 bp) and 3% for 07 genotypes (352, 454, 313, 328, 399, 408 and 475 bp). The others occur at a frequency < 2%.

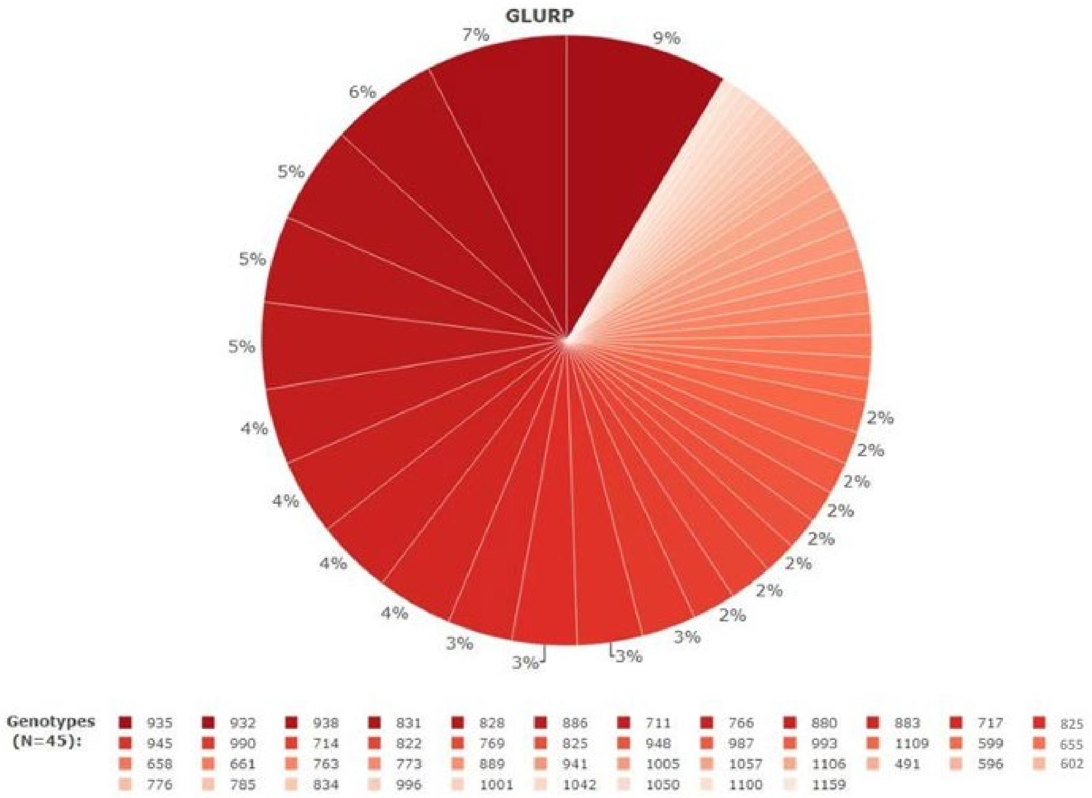

Figure S5. GLURP fragment sizes and frequencies. Forty-five (45) genotypes are identified based on the fragment sizes. From left to right, the genotypes are listed in order of decreasing frequency. A genotype 935 bp is the most abundant with a frequency of 9%. The frequencies are found at 7% for 01 genotype (932 bp), 6% for 01 genotype (938 bp), 5% for 03 genotypes (831, 828 and 886 bp), 4% for 04 genotypes (711, 766, 880 and 883 bp), 3% for 04 genotypes (717, 825, 945 and 990 bp) and 2% for 08 genotypes (714, 822, 769, 825, 948, 987, 993 and 1109 bp).The others occur at a frequency < 2%.

Table S1. Parasite density distribution by site among genotyped samples, N=157.

	**Total, N**	**Parasite density mean (parasites/μL blood)**	**95% CI**	**Z**	***P* value**
**Site**					
Urban	126	3721	1980.2-5461.8		
Rural	31	452.5	176.2-728.9	2.743	0.006

Table S2. MOI correlation with parasite density and age.

	**MOI**	***P* value**
Parasite density	r=0.3861	<0.001
Age	r= -0.0695	0.3868

Spearman's rank coefficient (r).
